# Development trend of urban design in “digital age”: Pan-dimensionality and individual-ubiquity

**DOI:** 10.1007/s11709-021-0735-7

**Published:** 2021-05-25

**Authors:** Jianguo Wang

**Affiliations:** School of Architecture, Southeast University, Nanjing, 210096 China

**Keywords:** digital age, urban design, multiple objectives, human-computer interaction, pan-dimensionality, individual-ubiquity

## Abstract

The wave of “digital age” featuring digital information is coming. Digital technology is profoundly changing the societal development direction and evolution paths. It also has significant bearing on production modes, social interactions and lifestyles. With regard to urban design, a system of knowledge about the creation and adaptation of material space forms that integrate humanities, art, technology and materials, digital technology has provided it with a brand-new and revolutionary scientific impetus for its evolution. The result of this evolution is “digital urban design paradigm based on human-computer interaction”, i.e., the urban development is moving toward “pan-dimensionality” and “individual ubiquity”. The future of urban design will construct a new approach to urban research and engineering, which is more complex, capable of accommodating and compatible with multiple goals of “instrumental rationality” and “value rationality”. Such a new approach shall be led by the probabilistic theory of “gray scale thinking”, reflecting quaternary synergetic view of “scientific rationality, ecological rationality, cultural rationality and technical rationality” to realize the cognitive progress of “engineering for the benefit of mankind”.

## 1 The advent of the “digital age”

Digital technology has already kicked off the fourth scientific and technological revolution. In the digital era of “everything is digital”, the multi-dimensional cognition of complex and macro urban systems and the inherent construction mechanism of urban physical forms have been interpreted more and more clearly, driven by high-speed mobile Internet, cloud computing, AI (Artificial Intelligence), ML (Machine Learning), IoT (Internet of Things), etc [1]. The digital technology and methods around the urban physical objects have also been dramatically developed. The possibility of integrating and even crystallizing mathematics with science, humanities and arts is increasing. Flaubert once said: “Science and art departed at the foot of the mountain and would reunite at the top”. Such a reunion has partially become a reality today.

**Technology development aims to reduce uncertain perceptions for people and for environment so as to achieve greater security and vision for a disorderly world.**

Newton and his contemporaries (Halley, Hooke, Boyle, etc.) created the “Mechanistic Theory”, which believed that everything in the world and its changes could be perceived by objective material facts. Henceforth, mankind no longer prostrated itself at the feet of the gods. Mechanistic Theory and the subsequent industrial revolution constructed a scientific cognitive world based on the continuity of the universe, believing that all things in the world can operate and evolve according to certain definite laws. The nineteenth-century theories of evolution, the doctrine of the cell, and the conservation of energy are the products of the continuum cognitive world. However, **the world is not as continuous or definite as wished in the development of the dimensions and scales of people’s cognition toward the world.** The scientific community developed from the Galileo transformation (i.e., simultaneous absoluteness) to the Lorenz transformation (i.e., simultaneous relativity), which solved and unified the

*Article history: Received Jan 20, 2021; Accepted Mar 21, 2021*

problem of the lack of a reference system in Maxwell's set of electromagnetic equations by assuming that the speed of light is constant, and time is variable. In 1900, Planck proposed that energy is part by part and cannot be divided (i.e., quantum), and started the modern physics. In 1905, Einstein found the "Special Theory of Relativity", in which he elaborated the photoelectric effect, quantum of light, wave-particle duality, and then Einstein and Bohr, Heisenberg, de Broglie, Schrödinger, and Dirac created the "Quantum Mechanics". This discovery partly appreciated Riemann geometry and his ideas. Most importantly, it rediscovered the uncertainty of the universe, and gradually unveiled the underlying logic of this world. Furthermore, it promoted a series of scientific progress and development, e.g., the theory of atomic nuclei with its military and commercial applications, solid state physics and related semiconductors and computer chips, the unveiling of cosmic and celestial bodies, etc.

**Digital technology can reduce uncertain perceptions for people and for environment, and raise certainty, security and anticipation fora disorderly world.**

Different from the historical industrial revolutions and associated "Mechanistic Theory", people have confronted a world of uncertainty, which is increasingly revealed by complex systems [2] since the beginning of last century. Although the universality and certainty of the physical world constructed by Newton partially disappeared with the rise of quantum mechanics, science has not abandoned its mission of "revealing objective laws". Continuity and certainty can help to reduce the scope and ranges of solutions at the expense of certain parameters, e.g., assuming that a law is true within a certain range provided that another parameter is invariant. The Turing revolution and related developments in information technology are the most fundamental scientific basis to confront an uncertain world. These consequent changes could lead to a brand new transmutation of civilization. In the past, people assumed that by discovering laws and following them, they could perceive the world, which was supposed to be continuous and certain. They could also eliminate their fear of the unknown and uncertainty. Whereas today we want to reduce the uncertain perception for people and for environment through information development, so that we can raise the certainty, security and anticipation for a disorderly world.

In recent years, Pan [3] put forward the concept of Artificial Intelligence (AI) 2.0 and expounded its differences from AI 1.0 in terms of demand problems, information environment, and target tasks. At the same time, he argues that the previous world was a dichotomy between the physical world and human society, while Cyberspace, a third element, also exists now. At the 2019 Annual Meeting of China's Urban Planning, Jianguo Wang suggested that urban vitality creation and enhancement requires "coexistence of explicit and implicit vitality", and addressed the issue of urban vitality regeneration. He also called for attention to both the past "face-to-face" physical presence of social interaction and to the currently increased digital "remote presence" (available at the website of WeChat Official Platform). Since the 1990s, with the improvement of computer computing power, human beings have entered the era of data explosion [4]. In terms of data size, for example, numerical simulations were measured in GB ($10^3$ MB) at the end of the 20th century, and it began to be measured in TB ($10^3$ GB) at the beginning of the 21st century, with many numerical simulations reaching the PB ($10^3$ TB) level and above in the last decade. In terms of data types, the growth and expansion of the Internet, the Internet of Things (IoT), the Internet products and the electronic devices have witnessed the generation of a large amount of structured and unstructured data such as web pages, images, audio, sensor time series, etc. We now have entered the digital age, or the era of big data from the perspective of scientific research and technological practice. The society with the vision of big data, pervasive computing, and the Internet of everything will show the important characteristics of ubiquity, intelligence, universality, and fineness [5].

## 2 Urban development trend in "digital age"–"pan-dimensionality" and "individual-ubiquity"

The wave of "digital era" featuring digital information is coming, and the Internet, big data, AI, IoT, and smart cities are profoundly changing the societal development direction and evolution paths, as well as human production modes, social interactions, and lifestyles [6,7]. In the digital age of "everything is digital", the once vertical urbane hierarchical structure is dissolving due to the driving force of high-speed mobile Internet, cloud computing and Internet of Things. Instead, the simultaneous, flat, discrete and non-hierarchical social structure is emerging. The multi-dimensional cognition of the complex urban mega-system and the inherent construction mechanism of the urbane physical forms are being interpreted more and more clearly. The digital technology approaches around the physical objects have been developed rapidly; the integration and even crystallization of mathematics and science, humanities and arts are becoming increasingly possible. In the context of the digital age, digital technology has provided a brand-new and revolutionary scientific impetus for the evolution of urban design discipline, and the result of this evolution is "digital urban design paradigm based on human-computer interaction". **In general, the urban development is showing two major trends. The first is from the "three-dimensional city" to the "pan-dimensional digital city"** (available at the website of Sohu). In the past, the narrow understanding of the city was mostly the physical space composed of length, width, and height, and sometimes the dimension of time was added in urban design and architecture. However, the city is a complex giant system, as it contains multi-source information from many fields such as society, economy, politics, culture and ecology, and presents the characteristics of pan-dimensionality. In the "algorithmic era", the construction of urban space form based on multi-source big data has the potential to obtain new ways of digital integration, cross-validation and application integration. The common dimensions can include human flow distribution based on LBS (location-based service) data, PoI (Point of Interest) business, public preferences based on social network platforms, and streetscape recognition based on artificial intelligence and machine learning, etc. **The second is from the city of "collective will" to the city of "individual ubiquity".** Individual ubiquity means that in the era of information technology, the large granularity crowd identification of the past (i.e., people were divided into groups) becomes today's identification of the massive individual-level granularity accuracy based on the Internet of everything along with visualization features. This makes it possible to identify and grasp the intrinsic mechanisms and characteristics of a city's development and evolution, and to better seek increasingly realistic solutions in a complex city.

## 3 Digital urban design paradigm based on human-computer interaction

"Digital urban design based on human-computer interaction" is the fourth generation paradigm of urban design in the context of the digital era (as shown in Fig. 1), which is a new digital urban design paradigm with the reconstruction of morphological integrity theory as the goal, human-computer interaction as the way, and the change of technical methods and tools as the core features [8].

### 3.1 Objective of fourth generation urban design paradigm: reconstruction of morphological integrity theory

Urban design aims to reveal the construction mechanism of "a-fruit-many-causes" urban form, and to shape the "four-phases-in-one" urban form and place environment with social, economic, ecological and cultural rationality.

In terms of engineering, it has always been the main measure dimension of success for being able to set the inductive recursive path of observing, analyzing, and addressing problems and to solve problems "higher, further, and stronger" in terms of engineering limits and threshold breakthroughs. Laboratories are getting larger and larger; devices are getting more and more sophisticated; measurements are getting more and more precise, and engineering design is mostly based on well-defined initial target methods. However, the evolution of human society today is increasingly characterized by the complexity, entirety, and "life community". Innovation lies in the connection of ideas, and isolated technologies may have limited future if lacking "outside help" to get them connected. In fact, the interconnection between the various subsystems of engineering design (e.g., transportation, industry, information, and ecology) is a manifestation of

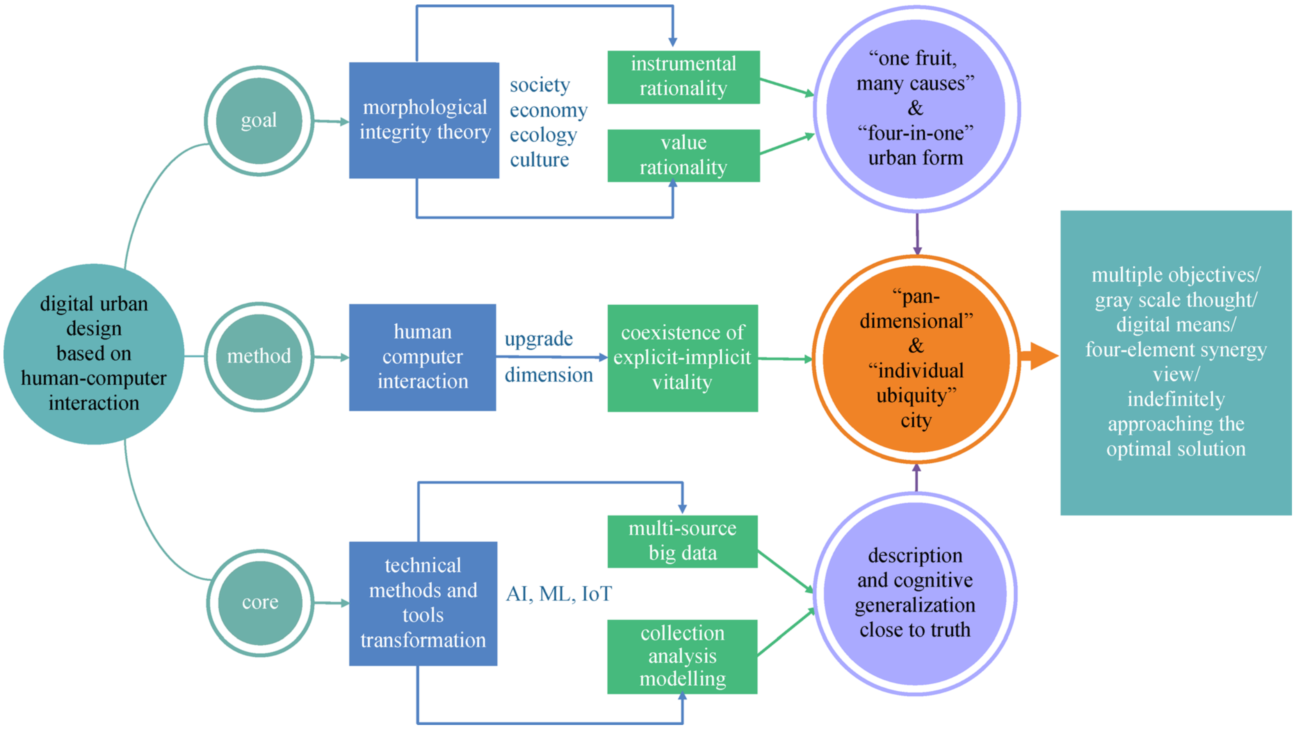

**Fig. 1** The flowchart of the digital urban design based on human-computer interaction.

grayscale thinking, which is the ability to see the world as having some middle ground, rather than merely seeing it as a black-and-white world. Engineering projects and their implementation are mostly of multi-sense and multi-functions with engineering and functional spillover effects as long as they have open social attributes and environment impacts., e.g., many bridges, hydroelectric facilities, etc. (such as Nanjing Yangtze River Bridge in China, Hoover Dam hydroelectric facility in the United States). Moreover, various transportation facilities in cities are not only solutions to the problems of convenience and accessibility of urban operations, but also part of the urban landscape, and signs of the city zoning (such as Beijing Inner Ring Elevated Line, LOOP in Chicago used to be the boundary of the central area of the city). In the 1920s, Le Corbusier's La Ville Radieuse had conceived a "domino" system of concrete frame structure, which can make the plan arrangement and facade treatment free. If we can further use flat plate structure, with more reinforcement inside the concrete but without beams or column caps, the building functions will be arranged and changed more flexibly. Although the structure does not conform to the most economical logic, this wins the flexibility of functional use and future variability from the perspectives of a longer structural lifespan.

China's reform and opening up and urbanization process in the past 40 years have achieved unprecedented social progress and swift economic take-off. It is the victory of successful use of "goal-oriented" and "efficiency-oriented" approach to achieve definite goals in the statistical sense. However, given the serious "urban diseases" in the urban construction over the years, it is fair to say that too much emphasis has been placed on the "economic rationality" of industrial development and that we have taken it as a measure of modernization and civilization evolution. "Cultural rationality" (social history, culture and arts) and "ecological rationality" (only one earth and a community of shared future) [8] are taken lightly because there is a problem with the understanding of "value rationality" [9]. Corbusier once believed in Descartes' thoughts and constructed a paradigm of "modern city" (Radiant City) under the background of scientific and technological development. However, this work ignored the value rationality of human society. Living crowds were simply viewed as abstract human beings or "human beings in groups", and the ultimate purpose of engineering construction was ignored. It is widely accepted that engineering construction should not only advance the scientific and technological progress, but also serve the social development, human welfare and the goal of "people-oriented". In the face of complex engineering research with social goals, it not only requires the support of continuous progress of science and technology to realize engineering and technical goals that can be clearly described and partially measured, but also needs rational value judgment from social, humanistic, ethical, ecological, and other dimensions [9].

### 3.2 Paradigm of the fourth generation urban design: human-computer interaction

The "invisible vitality" of urban dynamics is profoundly changing the principle of geographical location of the city [10]. Urban design used to focus on "the function, layout, space organization and operation and maintenance of physical space and entity form", i.e., "three-dimensional material entity + time dimension + static or sequential scene". Undoubtedly, urban design needs to analyze urban space objects with the help and application of engineering science and technical methods [11–13], including visual aesthetics, spatial experience, and place perception, etc., which require a knowledge base in the field of visual perceptual science, environmental behavior, and ergonomics etc. However, current urban design object can be changed from the static urban space in the past to the dynamic city with multi-dimensional composite, "individual as subject" or "individual ubiquity" of "human-centered" design. The construction of urban morphological mechanism and the way of place creation with precise scale and certain granular precision are surfacing.

**Creating and enhancing urban dynamism requires "coexistence of explicit and implicit vitality"**. The rapid growth and development of mobile Internet-based "implicit dynamics" is fundamentally changing the geographic location principles of previous urban planning and the way people interact with each other [14]. Today's smartphones have changed the underlying logic of society and life, i.e., the high integration of people and information or people having been digitized. The form and efficiency of social governance will undergo profound changes. Social cooperation model, refined social services (e.g., monitoring facilities of residential property can pay attention to various conditions of resident, access information, whether there are difficulties in special residents, prevention and control of COVID-19 [15]), and the way of information transmission are more vertical and flat. Great changes will also take place in telecommuting and online teaching and learning.

Today we are changing in the "digital revolution" or "algorithm era". Information of all kinds is being digitized and is the fundamental driving force behind the dramatic growth of cyberspace. The technology to analyze this vast amount of information is evolving rapidly, driven by artificial intelligence. We are also getting and referring to all kinds of information on cyberspace almost all the time, even being enveloped in a cloud of information. At the same time, practices are carried out in the real physical space. The convergence of physical space and cyberspace is advancing faster than expected. Obviously, the way people connect and the forms of society and economy are also changing dramatically [16].

**Understanding the city needs to upgrade its dimen-**

**sion and increase its resilience or robustness in development.** As Herbert Simon, a famous American scholar, pointed out, in the design of cities, the boundaries between the design of specific forms and the design of social systems have almost completely disappeared. Designing a city is designing a society. The main ways to deal with the uncertain urban development in the future are to seek a reasonable range of urban evolution range with appropriate boundary threshold (redundancy), and to cultivate the ability of self-organization and self-healing from the city’s bottom-up, by the cooperation of cross-domain, cross-industry and cross-border experts.

### 3.3 Core features of the fourth generation urban design paradigm: transformation of technical methods and tools

Digitalization is to convert many complex and variable information into numbers and data that can be measured, and then use these numbers and data to establish an appropriate digital model, transform them into a series of binary codes, and introduce them into the internal computer for unified processing, which is the basic process of digitization [17–19]. Digitalization focuses on results and organizes digitized information in an orderly way, which is convenient for enquiry retrieval, intelligent analysis and provision of solutions to relevant decision-making problems. Digitalization is an important concept unique to Chinese, and comes up naturally under the influence of the terms such as electronation, informatization, computerization and networking.

By collecting, analyzing, and integrating various social energy flows (ordered and disordered) [20], numerous individual-led social behavior big data, choice preference big data, and traffic information big data, etc., it is possible to see the relationship between urban social populations and changes in urban functional layout [21,22]. This is also an important scientific basis for large-scale urban design we do in cities today.

The data content includes space, format, activities, history, landscape, physical environment, energy consumption, etc. [23]. Specific work mainly includes data mining, data analysis and model construction, etc. Interdisciplinary expert cooperation has become an important impetus for urban design. In the past, professional design schemes and design drawings were mainly read by relevant professionals. However, today’s design results can be spread to the general public in a more efficient, intuitive (visual), concise and easy-to-understand way of theoretical expression [24].

For the first time in history, digital urban design has dramatically improved our ability to study and determine the causes and uncertainties behind the appearance of urban form with a high probability of convergence to correctness. Based on multi-source big data, machine learning and artificial intelligence [25–27], **urban design can already begin to explain the basic composition of urban space form elements, the holistic interpretation of social and population relations, the integration of urban vitality elements and their dynamic distribution and patterns**. In particular, it is possible to make more and more realistic and regular descriptions and generalizations of urban design objects that are interdisciplinary, cross-disciplinary, multi-scale, and beyond individual cognition and recognition abilities. It can also further reveal the direction of dynamic evolution of urban space forms.

## 4 Summary

Since the 19th century, we have been pursuing the universal values of evolution, Newtonian space-time framework mechanism, continuity and certainty. However, “rationalism” is not the whole issue of modernity. For example, what is “happiness”, “meaning”, and “equity”, and even how to achieve social justice and sustainable development per the United Nations paper, involve a series of social, humanistic, ethical, artistic and other disciplines. This clearly points to the diversity of value choices and differences in the hierarchy of demands, which cannot be explained and solved by simple scientific rationality. All this has been changed with the introduction of relativity, quantum mechanics and the second law of thermodynamics. Today and in the future, more and more uncertainties and the “Black Swan” have confirmed the universality of Heisenberg’s “Uncertainty Principle”.

The city is recognized as a complex giant system and even a cognitive “black box”. Urban design is a knowledge system related to the construction and adjustment of material space form integrating humanities, art, technology and materials [28]. Given the complex giant system of the city, to some extent, “uncertainty” is its underlying logic, and the increasingly deepening digital era should make it possible for us in the future to address the development and construction problems of this complex urban mega-system. Digital tools have greatly improved the usefulness of urban design, and a continuous convergence to the optimal solution is attainable.

The “Building Information Model” (BIM) of the construction industry integrates and further realizes the complex network-like connection of various related technical and professional systems [29], which can be dynamically modified and optimized, presenting the integrated optimization of buildings’ design, construction, operation and maintenance. Today, the city information model (CIM) associated with smart cities presents a mesh of urban construction systems and subsystems [30] “Roaming on the cloud, weaving a network under the cloud, connecting lines online, and nodes online” [31]. With the interaction of “points, lines, networks, and clouds”, cross-validation of information from different systems, and the use of iterative algorithms to obtain value domain optimization intervals with bottom-line thresholds, it is possible to construct a new idea of urban research and engineering construction that is more complex, which can accommodate and be compatible with multiple objectives of “instrumental rationality” and “value rationality”. And such a new approach is led by probabilistic “gray scale thinking”. The resulting urban design can reflect the quadratic synergistic view of “scientific rationality, ecological rationality, cultural rationality and technical rationality” to realize the cognitive progress of “engineering for the benefit of mankind”.

**Acknowledgements** The authors would like to acknowledge the financial support from the 13th Five Year National Science and Technology Support Key Project of China (No. 2017YPC0702500)

**Open Access** This article is licensed under a Creative Commons Attribution 4.0 International License (https://creativecommons.org/licenses/by/4.0/), which permits use, sharing, adaptation, distribution and reproduction in any medium or format, as long as you give appropriate credit to the original author(s) and the source, provide a link to the Creative Commons licence, and indicate if changes were made. The images or other third party material in this article are included in the article’s Creative Commons licence, unless indicated otherwise in a credit line to the material. If material is not included in the article’s Creative Commons licence and your intended use is not permitted by statutory regulation or exceeds the permitted use, you will need to obtain permission directly from the copyright holder. To view a copy of this licence, visit http://creativecommons.org/licenses/by/4.0/.
